# *Vibrio cholerae* ghosts (VCG) exert immunomodulatory effect on dendritic cells for enhanced antigen presentation and induction of protective immunity

**DOI:** 10.1186/s12865-014-0056-x

**Published:** 2014-12-31

**Authors:** Francis O Eko, Jayanti Mania-Pramanik, Roshan Pais, Qing Pan, Daniel M N Okenu, Arieian Johnson, Chris Ibegbu, Cheng He, Qing He, Raedeen Russell, Carolyn M Black, Joseph U Igietseme

**Affiliations:** Morehouse School of Medicine, Atlanta, GA USA; National Institute for Research in Reproductive Health, Mumbai, India; Clark Atlanta University, Atlanta, GA USA; Emory Vaccine Center, Emory University School of Medicine, Atlanta, GA USA; College of Veterinary Medicine, China Agricultural University, Beijing, 100094 China; Centers for Disease Control (CDC), Atlanta, GA USA

**Keywords:** VCG, BMDC, T-cell activation, *Chlamydia*, Immunity

## Abstract

**Background:**

We previously showed that the *Vibrio cholerae* ghost platform (VCG; empty *V. cholerae* cell envelopes) is an effective delivery system for vaccine antigens promoting the induction of substantial immunity in the absence of external adjuvants. However, the mechanism by which these cell envelopes enhance immunity and stimulate a predominantly Th1 cellular and humoral immune response has not been elucidated. We hypothesized that the immunostimulatory ability of VCG involves dendritic cell (DC) activation.

**Objective:**

The aims of this study were: a) to investigate the ability of DCs [using mouse bone marrow-derived DCs (BMDCs) as a model system] to take up and internalize VCGs; b) to evaluate the immunomodulatory effect of internalized VCGs on DC activation and maturation and their functional capacity to present chlamydial antigen to naïve and infection-sensitized CD4+ T cells and; c) to evaluate the ability of VCGs to enhance the protective immunity of a chlamydial antigen.

**Results:**

VCGs were efficiently internalized by DCs without affecting their viability and modulated DC-mediated immune responses. VCG-pulsed DCs showed increased secretion of proinflammatory cytokines and expression of co-stimulatory molecules associated with DC maturation in response to stimulation with UV-irradiated chlamydial elementary bodies (UV-EBs). Furthermore, this interaction resulted in effective chlamydial antigen presentation to infection-sensitized but not naïve CD4+ T cells and enhancement of protective immunity.

**Conclusions:**

The present study demonstrated that VCGs activate DCs leading to the surface expression of co-stimulatory molecules associated with DC activation and maturation and enhancement of protective immunity induced by a chlamydial antigen. The results indicate that the immunoenhancing activity of VCG for increased T-cell activation against antigens is mediated, at least in part, through DC triggering. Thus, VCGs could be harnessed as immunomodulators to target antigens to DCs for enhancement of protective immunity against microbial infections.

## Background

*Chlamydia trachomatis* is a major cause of sexually transmitted infection, which if untreated often lead to severe complications, such as pelvic inflammatory disease, ectopic pregnancy and tubal infertility [1,2] A vaccine to prevent chlamydial infection is urgently needed but none currently exists and most experimental *Chlamydia* vaccines have so far yielded inadequate protection [3]. This failure to afford sterilizing immunity may partly be due to their inability to induce a favorable immunostimulatory and cytokine environment. Immunomodulators (adjuvants) act by activating the innate immune system to provide an adequate co-stimulatory and favorable cytokine environment to support the stimulation of the desired adaptive immune responses.

Many of the conventional vaccines in use today are made of attenuated or killed pathogens, thus containing naturally a number of signals able to activate the innate immune response [4,5]. Subunit vaccines on the other hand rely on the incorporation of effective adjuvants to modulate immunity [6]. Although many substances are under investigation for their effectiveness as adjuvants, none is currently approved for human use [7]. The FDA approved Aluminum salts (alum) adjuvants induce mainly humoral immunity and is not very effective at enhancing cell-mediated immune responses [8,9]. There is thus a need to develop new and effective adjuvants that can be used in humans.

*Vibrio cholerae* ghosts (VCGs) are empty bacterial cell envelopes devoid of cytoplasmic contents and cholera toxin and are generated by the genetic inactivation of *Vibrio cholerae* cells that results in expulsion of cytoplasmic contents [10]. VCGs share the functional and antigenic determinants of the envelope with their living counterparts [10]. They are attractive for use, as non-living vaccine delivery vehicles because they are non-toxic, maintain the structural and functional integrity of expressed antigens, and are excellent for delivery of vaccine antigens to primary antigen-presenting cells [11]. We previously showed VCG-based subunit vaccines induced significant chlamydial immunity in the absence of external adjuvants suggesting VCGs possess potent adjuvant properties [12,13]. However, the mechanism of VCG-mediated immune enhancement has not been delineated. We hypothesized that the immunostimulatory ability of VCG is exerted via dendritic cell (DC) activation. DCs are the most potent antigen presenting cells (APCs) and efficiently acquire and present antigens to stimulate adaptive immunity [14] through expression of a combination of cell surface and secreted molecules that influence the type of immune response stimulated. Secretion of T-helper type-1 (Th1)-driving cytokines, such as interleukin-12 (IL-12), by DCs favors induction of Th1-biased responses. In this regard, we previously showed that *Chlamydia*-pulsed IL-10-deficient DCs produced sterilizing, long-term chlamydial immunity following adoptive immunization of mice indicating that they possessed the necessary antigenic, costimulatory and immunomodulatory machinery for inducing an optimal protective immunity [15].

To clarify the cellular and molecular basis of the immunomodulatory action of VCGs, the present study will: a) investigate the ability of mouse bone marrow-derived DCs (BMDCs) to take up and internalize VCGs; b) determine the effect of internalized VCGs on DC activation, maturation and differentiation and; c) evaluate the immunomodulatory ability of VCGs to enhance the functional capacity of BMDCs to present chlamydial antigen to naïve and infection-sensitized CD4+ T cells and stimulate T cell proliferation. We show that VCGs were efficiently internalized by DCs without affecting their viability. Also, VCG-pulsed DCs showed increased secretion of proinflammatory cytokines and expression of co-stimulatory molecules associated with DC maturation following stimulation with UV-EBs. Furthermore, this interaction resulted in effective chlamydial antigen presentation to infection-sensitized but not naïve CD4+ T cells, as indicated by enhanced T cell proliferation and secretion of Th1-type cytokines.

## Methods

### *Chlamydia* stocks and antigens

Stock preparations of *C. trachomatis* serovar D strain were generated by propagating elementary bodies (EBs) in HeLa cells as previously described [16]. All stocks were titrated on HeLa cell monolayer’s followed by purification of EBs over renografin gradients [16] and stored at −70°C. Chlamydial antigens were prepared by UV-inactivation of EBs for 3 h and stored at −70°C.

### Mice

All mice used in these studies were of the C57BL/6 strain (female, aged 6 to 8 weeks) from The Jackson Laboratory (Bar Harbor, ME). They were housed in the animal facility of Morehouse School of Medicine (MSM) and animal study protocols were performed in compliance with Institutional Animal Care and Use Committee (IACUC) (IACUC protocol #09-18) and prescribed federal guidelines.

### Production of *Vibrio cholerae* ghosts (VCG)

Production of VCG was carried out essentially as described previously [10]. Briefly, competent *Vibrio cholerae* H1 cells harboring the lysis plasmid pDKLO1 were grown in brain heart infusion medium (BHI) at 37°C to mid-log phase. Cell lysis was achieved by the addition of 3-methyl benzoate to induce gene *E* expression from the Pm promoter, which results in rapid de-repression and protein E-mediated lysis. The extent and rate of lysis was followed by measuring the absorbance of the lysing culture at 600 nm and quantified as decrease in turbidity per unit of time. At the end of lysis cultures were harvested, washed with phosphate-buffered saline (PBS) or a low ionic buffer and lyophilized. Lyophilized VCGs were weighed and the number of CFU per milligram of VCG was estimated based on the total number of CFU in the culture medium at the highest absorbance attained before lysis. Ghost preparations were stored at room temperature until use.

### Dendritic cell (DC) isolation and culture

DCs were generated from the bone marrow (BMDC) of 6 week-old C57BL/6 mice as described previously [17] using IL-4 and GM-CSF. Purified DCs at a concentration of 5 × 10^5^ cells/ml were incubated with 100 μg of VCG or UV-EB (at an MOI of 10) in 24-well for 24 h. Culture supernatants were collected and assayed for cytokines (IL-12, IL-10, IL-4 and TNF-α) and harvested cells were stained for flow cytometric analysis.

JAWS II cells (ATCC, CRL-1194; Manassas, VA), an immortalized immature dendritic cell line, which was established from bone marrow cells of a p53-knockout C57BL/6 mouse [18] were used for VCG uptake experiments. Cells were cultured in complete culture medium consisting of IMDM with 10% FCS, 4 mM L-glutamine, 10 U/ml penicillin and 100 μg/ml streptomycin, 0.5 mM 2-ME, 1 mM sodium pyruvate, and 5 ng/ml murine GM-CSF essentially as described [19].

### Uptake of VCG by DCs and confocal microscopy

VCGs were labeled using Alexa Fluor® 594 Protein labeling kit (Life Technologies, Grand Island, NY) according to the manufacturer’s instructions with minor modifications. Briefly, the lyophilized VCGs resuspended in PBS were transferred to the vial of reactive dye and the reaction mixture was stirred for 1 hour at room temperature. Following the conjugation reaction the unbound dye was washed off via centrifugation instead of passing through the gel filtration column provided for soluble proteins. JAWS II cells (5 × 10^5^ cells/ml) were plated on Lab Tek II Chamber slides (Thermo Scientific, Pittsburgh, PA) and incubated with 100 mg/ml of Alexa 594-labeled VCG for 45 min at 37°C/5% CO_2_ to measure specific uptake. After washing three times with ice cold PBS containing 2% BSA (PBS-BSA) to remove free non-internalized VCG, the cells were fixed in cold acetone for 10 min at room temperature and again washed three times with PBS-BSA. The cells were then incubated for 1 h with biotin-labeled anti-mouse CD11c antibodies and streptavidin-conjugated Alexa 488 (Life Technologies, Grand Island, NY) for 1 h. Following washing with PBS-BSA, the slides were mounted in a drop of Vectashield medium (Vector Labs, Burlingame, CA). A Leica TCS SP5 Confocal Microscopy System (Leica Microsystems; Bannockburn, IL) equipped with a 40× and 63×/1.40 NA oil-immersion objectives were used to scan the slides and then analyzed using the Leica Application Suite, Advanced Fluorescence (LAS AF) and Image J softwares.

### Assessment of DC viability

To assess the viability of BMDCs after incubation with VCGs, the DCs were pulsed with VCG for 2 h, washed and incubated further for 24 h and viability was assessed using the XTT cell viability/proliferation assay according to the manufacturer’s instructions (Cell Signaling Technology, Danvers, MA). BMDCs (2 × 10^4^/well) were incubated with 50 or 100 mg of VCGs/ml at 37°C for 2 h. The DCs were then washed to remove excess VCGs followed by incubation in complete culture medium. After 24 h, premixed XTT reagent was added to each well and cells were incubated for an additional 4 h at 37°C. The absorbance was measured at 450 nm using a Softmax plate reader after thoroughly shaking the plate for 60 sec. The cytotoxic agent Triton X-100 (0. 5%) was used as a control.

### Fluorescent antibody staining and flow cytometry

Harvested BMDCs pulsed with VCG or UV-EB were stained with monoclonal antibodies against CD11c, CD83, CD86, CD14 or CD40, CD80, 1Ab, and CD71 conjugated with either PE- or FITC (Pharmingen, San Diego, CA) and analyzed by flow cytometry on a FACScan Flow Cytometer (Becton-Dickinson, CA). Controls were incubated with isotype-matched irrelevant antibodies. Staining was carried out in PBS containing 5% FCS. Marker expression was assessed on gated CD11c cells.

### Assessment of antigen-specific CD4+ T cell responses

Splenocytes obtained from spleens of naïve and immune mice (mice previously infected intranasally with 1 × 10^5^ IFU of live *Chlamydia*) using the gentleMACS Dissociator (Miltenyi Biotech, Auburn, CA) were resuspended in PBS containing BSA and EDTA. CD4+ T cells were then purified by positive selection using the MidiMACS system and labeled CD4 (L3T4) mouse microbeads (Miltenyi Biotech, Auburn, CA). CD4+ T cell purity was at least >95% by flow cytometry. BMDC (2 × 10^5^ cells/well) pulsed with UV-EB in the presence or absence of VCG for 24 h were cultured with naïve or immune CD4^+^ T cells at 2 × 10^5^ cells/well. Control cultures contained BMDCs and naïve T cells. After 48 h, supernatants were harvested and assayed for Th1/Th2 cytokines using the Bio-Plex cytokine assay kit in combination with the Bio-Plex Manager software (Bio-Rad, Hercules, CA). The mean and SD of all replicate cultures were calculated. The experiment was repeated three times.

### Measurement of T cell proliferation

Purified *Chlamydia*-induced immune CD4^+^ T cells (2 × 10^5^ cells/well) were cultured in 96-well plates (Corning Glass Work, Acton, MA) with 2 × 10^5^ cells/well of BMDCs alone or BMDCs previously pulsed for 24 h with VCG in 200 μl of C-RPMI medium supplemented with GM-CSF in the presence of chlamydial antigen (10^6^ IFU/ml UV-irradiated EBs) at 37°C in 5% CO_2_. After three days, proliferation was assessed by the 5-Bromo-2′-deoxy-uridine (BrdU) cell proliferation assay described previously [20] according to the manufacturer’s instructions (Roche Molecular Biochemicals, Indianapolis, IN). T cells cultured in the absence of chlamydial antigen served as internal control.

### Adoptive immunization, challenge and analysis of protective immunity

Dendritic cells (JAWS II cells; 1×10^6^ cells/ml) were incubated with MEM medium containing L-glutamine, ribonucleosides and deoxyribonucleosides (HyClone, Logan, Utah) supplemented with 5 ng/ml murine GM-CSF (MEMS), UV-EB (1×10^6^ IFU/ml) or UV-EB + VCG (100 μg/ml) at 37°C. After 24 h, the UV-EB or UV-EB + VCG-pulsed DCs were washed three times with cold PBS and injected subcutaneously into mice twice, 2 weeks apart at a concentration of 1×10^6^ cells/mouse in 25 μl PBS. Six days before challenge, mice were treated with 2.5 mg/mouse Depo-Provera (Pfizer/UpJohn Co., Kalamazoo, MI) to synchronize estrous and challenged intravaginally 2 weeks after the last immunization with 5 × 10^4^ IFU of *C. trachomatis* serovar E in 5 μl of SPG buffer. To assess the level of infection, cervicovaginal swabs collected every 3 days following challenge were incubated on HeLa cell monolayers grown on glass cover slips in 24-well plates. Cells were fixed with 2% paraformaldehyde and stained using a genus-specific rabbit monoclonal antibody and FITC labeled goat anti-rabbit secondary antibody. The number of inclusions was counted under an Olympus B-Max microscope in 10–12 random fields. The mean ± SD of recovered chlamydial IFU per group was calculated.

On day 24-post challenge, serum samples were collected by heart bleed and total T cells were purified from the spleens of challenged mice.

### Detection of antibody isotype levels by ELISA

Blood samples were collected by heart puncture and vaginal lavage was obtained by flushing the vaginal vault with 100 μl of PBS on day 24 after challenge. The amount of specific antibodies (IgG2a and secretory IgA) in sera and vaginal washes against chlamydial antigen was measured by a standard ELISA procedure described previously [21]. Plates were incubated with HRP-conjugated goat anti-mouse IgA, IgG1or IgG2c isotype (Southern Biotechnology Associates, Inc., Birmingham, Ala.) for 1 h and developed with (3,3′,5,5′-tetramethylbenzidine) (TMB). The optical density was measured at 450 nm on a Microplate reader. Results, generated simultaneously with a standard curve, display data sets corresponding to absorbance values as mean concentrations (ng/ml) ± SD and represent the mean values from triplicate experiments.

### Statistical analysis

Statistical analyses were performed with the GraphPad Prism package (GraphPad Software, Inc. La Jolla, CA, USA) on a Macintosh computer. The statistical significance of the difference between two groups was evaluated by Student’s t-test and between more than two groups by one-way ANOVA. Differences were considered to be significant at p* < 0.05 or p** < 0.01.

## Results

### JAWS II DCs efficiently phagocytose (internalize) VCGs

**Figure 1:**
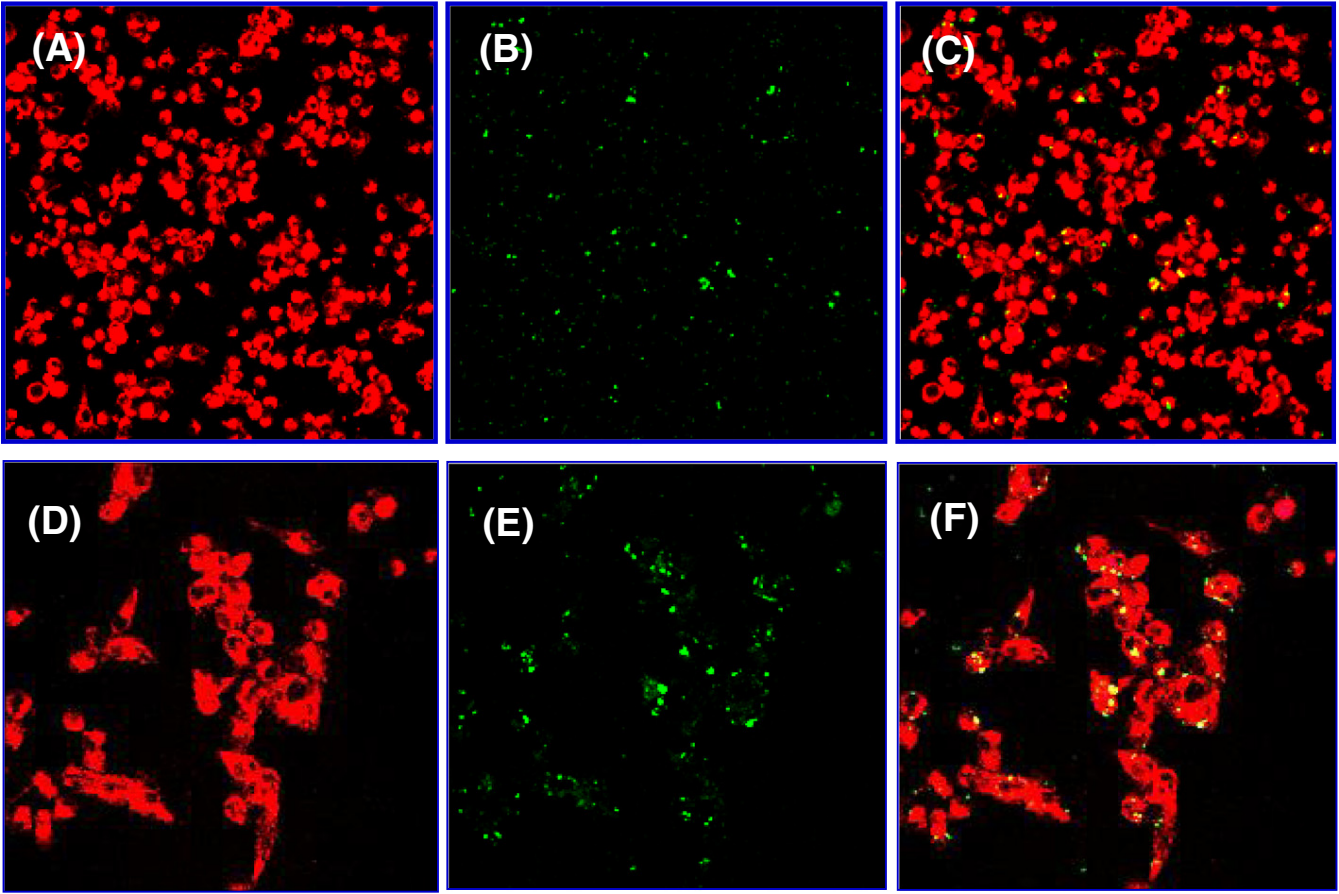
**Uptake and internalization of VCGs by JAWS II dendritic cells (DCs).** Internalization of fluorescence-labeled VCGs by JAWS II DCs and localization of VCGs within DCs were visualized by confocal scanning microscopy. DCs were incubated on Chamber slides for 45 min with 100 μg/ml of Alexa 594-labeled VCGs. Following extensive washing with PBS-BSA, cells were fixed in cold acetone and incubated with biotin-labeled anti-mouse CD11c antibodies and streptavidin-conjugated Alexa 488. Images were captured with a Leica scanning microscope with a 40x **(A-C)** or 63x oil **(D-F)** objective and each image is a representative of a single z-stack of various optical sections. **(A & D)** Alexa 488-labeled DCs, **(B & E)** Alexa 594-labeled VCGs, and **(C & F)** internalized VCGs shown as yellow or greenish yellow particles, which represent direct overlay of green and red fluorescent structures. Alexa 488-labeled cells and Alexa 594-labeled VCGs are shown in pseudo red and green colors, respectively.

The ability of JAWS II DC to internalize VCG was investigated by culture of Alexa 594 (red)- labeled DCs with Alexa 488 (green)-labeled VCG. Before incubation with antibodies, DC + VCG cultures were washed several times by centrifugation at 300 *g* to remove non-internalized VCG and ensure that only the cell-associated ones are retained. The presence of internalized Alexa 594-labeled VCG was captured on a Leica scanning confocal microscope with a 40× (A-C) or 63× oil (D-F) objective (Figure 1). VCGs were efficiently internalized following co-incubation with DCs for 45 min (Figure 1C & F). However, detectable internalization was observed as early as 10 min after co-culture of VCG with the DC cultures (data not shown).

### VCG have no cytotoxic effect on cultured BMDCs

**Figure 2:**
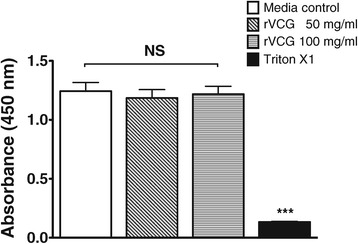
**Effect of VCGs on dendritic cell viability.** The viability of BMDCs after incubation with VCGs was assessed using the XTT cell viability/proliferation assay. BMDCs cultured in 96-well flat-bottom plates (2 × 10^4^/well, BD Biosciences) were incubated with 50 μg/ml or 100 μg/ml of VCGs for 2 h. The XTT cell viability assay was performed 24 h later to investigate the toxicity of VCGs on BMDCs. Results are expressed as absorbance values of VCG-pulsed cells relative to media alone and the bars represent the mean and S.D. of three independent experiments. Significant difference between cultures incubated with media alone or cultures incubated with VCGs and Triton X-100 is indicated by asterisk (P** <0.001).

The effect of internalized VCG on the viability of BMDCs was assessed after incubation of BMDCs with 50 μg and 100 μg of VCG for 2 h. The nonradioactive XTT colorimetric assay was performed 24 h later to investigate possible toxic effects of VCGs on DCs. The results showed VCG had no cytotoxic effects on the viability of BMDCs at both concentrations (Figure 2). No significant differences in viability of BMDCs incubated with and without VCG were detected. In contrast, there was a significant decrease in the viability of BMDCs incubated under the same conditions with the cytotoxic agent Triton X-100.

### VCG enhanced the activation and maturation of BMDCs

**Figure 3:**
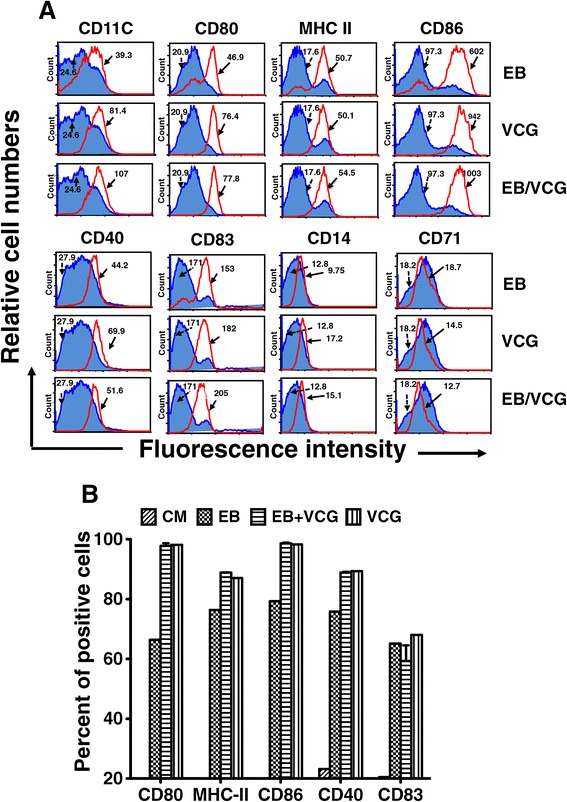
**Effect of VCG on activation marker expression by BMDCs using FACS analysis.** Bone marrow-derived DCs (BMDCs) were isolated from mice by established procedures using IL-4 and GM-CSF and characterized as loosely adherent mononuclear cells expressing high levels of CD11c but lacking B220 surface antigens. Harvested cells were pulsed with VCGs or UV-EBs or UV-EBs + VCGs for 24 h, stained with conjugated monoclonal antibodies against CD11C, CD40, CD80, CD83, CD86, 1Ab, CD14 and CD71 or isotype-matched controls, and quantified in triplicate by flow cytometry. The data shows the mean fluorescence intensity of staining **(A)** and percentage of cells **(B)** expressing the indicated marker after treatment with antigens (unshaded area) or culture media (Blue shaded area). Data is from one of two experiments with similar results.

Purified BMDCs were pulsed *ex vivo* either with UV-irradiated chlamydial EBs (UV-EBs) or VCG or UV-EBs together with VCG or culture medium alone (CM, control) for 24 h and changes in surface activation marker expression, including CD40, CD80, CD83, CD86 and MHC II (IAb) were assessed by FACS analysis. Figure 3A shows that following overnight incubation, UV-EBs up-regulated the expression of co-stimulatory molecules involved in DC activation and maturation, including CD80, CD86, MHC class II, CD40 and CD83. This up-regulation was further enhanced following co-incubation with VCGs or VCG alone. In contrast, incubation of DC with antigens resulted in similar or decreased expression of CD14 and CD71 compared to incubation with CM alone. Figure 3B shows the percentage number of cells positive for the indicated markers following incubation with antigens or CM. These results reveal that pulsing of BMDCs with the antigens lead to development of the DC into a mature phenotype as indicated by enhancement of expression of the co-stimulatory molecules associated with DC activation and maturation.

### VCG enhanced the production of Th1 promoting cytokines by BMDCs

**Figure 4:**
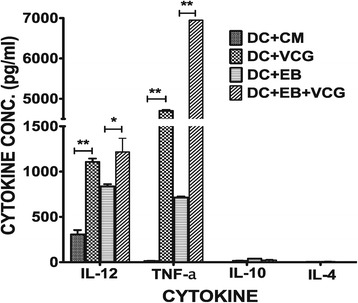
**Effect of VCG on the production of Th1/Th2 promoting cytokines by BMDCs.** BMDCs were pulsed *ex vivo* with UV-EBs, UV-EB + VCG, or medium for 24 h. Culture supernatants were collected and assayed for IL-12, TNF-α, IL-10 and IL-4 cytokines by cytokine ELISA using the Bio-Plex cytokine assay kit in combination with the Bio-Plex Manager software. The concentration of the cytokines in each sample was obtained by extrapolation from a standard calibration curve generated simultaneously. Data are shown as the mean values (± S.D.) for triplicate cultures for each experiment. The results are from two independent experiments. Statistically significant differences between DCs alone and treatment groups was evaluated at (p* < 0.05) or (p** < 0.01).

Culture supernatants of BMDCs pulsed *ex vivo* with UV-EBs, UV-EB + VCG, or medium for 24 h were assayed for the production of Th1/Th2-promoting cytokines (IL-12, IL-10 and IL-4) as well as TNF-α by cytokine ELISA and the results are shown in Figure 4. BMDCs pulsed with VCG or UV-EBs displayed significantly (*p* ≤0.01) increased levels of IL-12 and TNF-α but not IL-10 and IL-4 compared to BMDCs pulsed with medium alone (Figure 4). Levels of IL-12 and TNF-α secreted were significantly (*p* ≤0.01) enhanced when BMDCs were pulsed with UV-EBs + VCG. These results suggest that VCGs enhanced the production of Th1 promoting cytokines by BMDCs are thus capable of priming a robust Th1 response.

### VCG enhanced BMDC presentation of chlamydial antigen to immune CD4+ T cells

**Figure 5:**
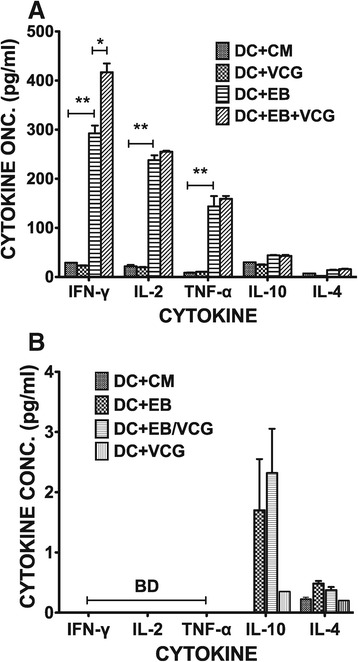
**Effect of VCG on BMDC presentation of chlamydial antigen to immune and naïve CD4+ T cells.** BMDC (2 × 10^5^ cells/well) pulsed for 24 h with UV-EB in the presence or absence of VCG were cultured with purified immune **(A)** or naïve **(B)** CD4^+^ T cells (2 × 10^5^ cells/well) for 48 h. Control cultures contained BMDCs and purified T cells without antigen (DC alone; DC+CM). At the end of the incubation period, supernatants were harvested and assayed for Th1 and Th2 cytokines, using the Bio-Plex cytokine assay kit in combination with the Bio-Plex Manager software. The concentration of the cytokine in each sample was obtained by extrapolation from a standard calibration curve generated simultaneously. Data were calculated as the mean values (± S.D.) for triplicate cultures for each experiment. The results are from two independent experiments. Significant differences between DCs alone and treated DCs and between DCs pulsed with UV-EB with and without VCG are indicated by two asterisks (*P* <0.01) and an asterisk (*P* <0.05) respectively.

To assess the effect of VCG on the capacity of BMDCs to process and present antigen to T cells, BMDCs pulsed with UV-EBs with and without VCG for 24 h were co-cultured with splenic CD4+ T cells from immune and naïve mice for 48 h and the amount of secreted cytokines was measured by multiplex ELISA assay. Supernatants from BMDC cultures incubated with immune CD4+ T cells produced significantly (*p* ≤0.01) higher amounts of IFN-γ (a Th1-type cytokine) compared to IL-4 (a Th2 cytokine) (Figure 5A). Importantly, the amount of IFN-γ produced by CD4+ T cells restimulated with UV-EBs in the presence of VCG was significantly (*p* ≤0.05) higher than that incubated with UV-EBs alone. On the other hand, incubation of naïve CD4+ T cells with BMDCs pulsed with UV-EBs in the presence or absence of VCG did not produce measurable amounts of cytokines (Figure 5B). Also, CD4+ T cells incubated with BMDCs in the absence of chlamydial antigen did not produce appreciable levels of cytokines (Figure 5). These results indicate that chlamydial antigen presentation to immune CD4+ T cells by BMDCs is enhanced by VCG, leading to the production of significantly (*p* ≤0.05) higher levels of IFN-γ.

### VCG enhanced the proliferation of immune CD4+ T cells in response to restimulation by UV-EBs

**Figure 6:**
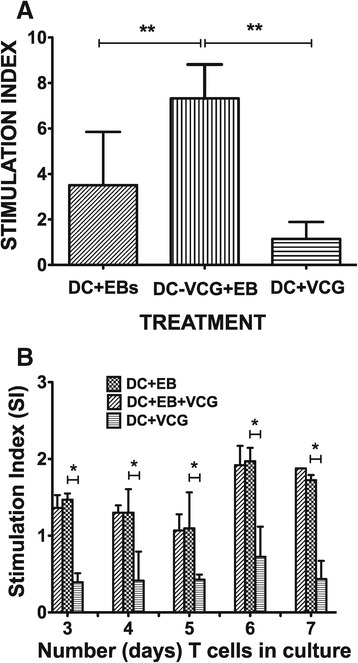
**Effect of VCG on the proliferation of immune or naïve CD4+ T cells.** Purified *Chlamydia* sensitized CD4^+^ immune **(A)** or naïve **(B)** T cells were cultured with BMDCs or VCG-pulsed BMDCs for 24 h and restimulated *in vitro* with chlamydial antigen (10^6^ IFU/ml UV-irradiated EBs) for 3 days. The antigen-specific proliferative response was determined using the BrdU incorporation assay; incorporation was detected by addition of ABTS substrate and the optical density was read at 405 nm using a scanning multi-well spectrophotometer. Results are expressed as the stimulation index (SI), the ratio between absorbance values of stimulated and non-stimulated cells and the bars represent the mean and S.D. of three independent experiments. **p < 0.001 (BMDCs versus BMDCs + EBs; BMDCs + EBs + VCG).

Immune CD4 T cells were incubated with BMDC pulsed with either UV-EB or UV-EB + VCG or VCG and T cell proliferation was assessed by the 5-Bromo-2′-deoxy-uridine cell proliferation assay. The mean absorbance values were 0.8800 ± 0.0820 (T cells + UV-EB) 1.837 ± 0.052 (T cells + UV-EB + VCG); 0.2875 ± 0.026 (T cells + VCG) and 0.25070 ± 0.0350 (T cells without UV-EB, control)]. To determine the effect of VCG on specific T cell proliferation, we analyzed stimulation index (SI) values (the ratio between absorbance values of stimulated and non-stimulated cells) obtained after stimulation of CD4+ T cells with chlamydial antigen (UV-EB) in the presence or absence of VCG. The derived SI values are shown Figure 6A. The results show that CD4+ T cells cultured with UV-EB + VCG proliferated significantly higher (p < 0.01) than those cultured with UV-EB alone. In contrast, T cells cultured with VCG without chlamydial EBs did not proliferate significantly.

We further investigated if VCG could also influence the ability of naïve CD4 T cells to proliferate in the presence of chlamydial antigen (UV-EB). Thus, naïve CD4 T cells were incubated with BMDC and either UV-EB or UV-EB + VCG or VCG alone and T cell proliferation was assessed by the XTT cell proliferation assay. Derived SI values calculated from the mean absorbance values are shown in Figure 6B. The results showed that unlike immune CD4 T cells, naïve CD4 T cells did not proliferate significantly until day 6 following stimulation with the various antigens. In contrast with immune T cells, VCG had no effect on the ability of T cells to proliferate following stimulation with UV-EB. As expected, naïve T cells cultured with VCG without chlamydial EBs did not proliferate significantly. The results indicate that VCG enhanced BMDC-mediated chlamydial antigen presentation to immune but not naïve CD4+ T cells as measured by T cell proliferative responses.

### VCG enhanced the specific immune responses elicited after challenge of immunized mice

**Figure 7:**
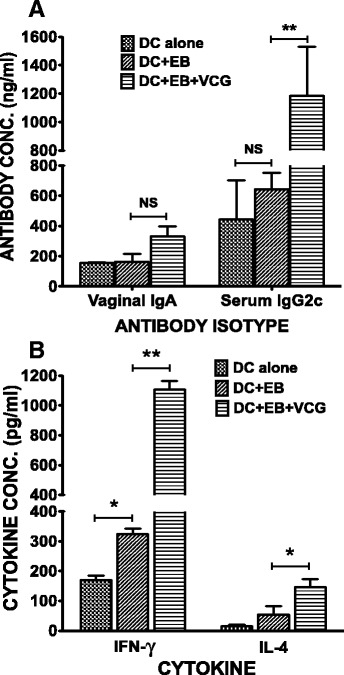
**Memory antibody and Th1/Th2 cytokine responses elicited 24 days postchallenge.** Groups of mice were adoptively immunized and challenged intravaginally as described in the materials and methods section. About 24 days postchallenge, serum, vaginal lavage and spleens were collected. The amount of memory antibodies **(A)** elicited in genital lavage and serum was assessed by an antibody ELISA. Results generated simultaneously with a standard curve display data sets corresponding to absorbance values as mean concentrations (ng/ml) ± SD of triplicate cultures for each experiment. The results are from two independent experiments. *Statistically significant (*p* < 0.05) differences between DC + EB and DC + EB + VCG groups. Also, purified splenic T cells were restimulated *in vitro* with UV-irradiated chlamydial EBs and IFN-γ and IL-4 production were assessed as a measure of Thl and Th2 responses, respectively. The amount of cytokine contained in supernatants of culture-stimulated cells was measured by single cytokine ELISA. The concentration of the cytokine in each sample was obtained by extrapolation from a standard calibration curve generated simultaneously. Data were calculated as the mean values (± S.D.) for triplicate cultures for each experiment **(B)**. The results are from two independent experiments. *Statistically significant (*p* < 0.05) differences between DC + EB and DC + EB + VCG groups.

The induction of antigen-specific antibody and cytokine responses was evaluated 24 days after challenge of adoptively immunized mice. Mice immunized with the DC + UV-EB + VCG elicited significantly higher (*P* < 0.05) levels of serum IgG2c antibodies compared to DC + UV-EB or DC controls. Although vaginal IgA levels elicited in the presence of VCG were not significantly different from the groups immunized without it, its presence showed a clear immunogenic advantage (Figure 7A). Total T cells purified from the spleen were cultured with γ-irradiated APCs and stimulated *in vitro* with UV-EBs (chlamydial antigen). IFN-γ and IL-4 production in supernatant cultures were measured by cytokine ELISA. The results showed that mice vaccinated with DC + UV-EB + VCG produced significantly (*p* ≤0.05) higher chlamydial-specific IFN-γ (1107.107 ± 58.67 pg/ml) compared to mice immunized with DC + UV-EB (324.273 ± 17.39) or DCs alone (169.857) (Figure 7B).

### VCG enhanced the ability of adoptively immunized mice to resolve a chlamydial genital infection

**Figure 8:**
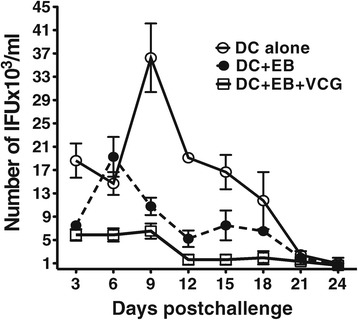
**Effect of VCG on reduction of IFU in adoptively immunized mice.** Immunized mice were challenged intravaginally with 5 x 10^4^ IFU of live chlamydiae 24 days after the last immunization. One week prior to challenge, mice were administered Depo Provera to stabilize the estrous cycle and facilitate a productive infection. Infections were monitored by cervicovaginal swabbing of individual animals every 3 days and *Chlamydia* was isolated from swabs in tissue culture and enumerated. The data show the mean recoverable IFUs ± S.D. The experiment was repeated to contain 6 mice per group.

Immunized mice were challenged intravaginally with live *Chlamydia* 24 days after the last immunization and periodically monitored for clearance. Figure 8 shows that mice that received DC + UV-EB + VCG shed fewer chlamydial IFUs after intravaginal challenge with live *Chlamydiae* compared to mice that received DC alone or cells without VCG.

## Discussion

The novel VCG platform is an effective carrier/delivery system for vaccine antigens, promoting the induction of significant immunity in the absence of external adjuvants, suggesting that VCGs possess potent immunostimulatory/adjuvant properties [12,13,22]. We hypothesized that the immunostimulatory ability of VCG is exerted via DC activation. DCs are potent antigen presenting cells that are essential for the initiation of primary immune responses, especially those essential in priming the differentiation of naïve T cells to Th1 or Th2 sub-types [23,24]. Efficient antigen uptake by antigen presenting cells (APCs), especially by professional APCs such as DCs, processing and presentation is a prerequisite for directing T- and B-cells towards the desired immune responses. The phenotypic and functional characteristics of DCs upon antigen uptake are intimately linked to their stage of maturation. Thus, upon activation and maturation, antigen-loaded DCs initiate antigen-specific immunity leading to T cell proliferation and differentiation into helper and effector cells with unique functions [25]. To analyze the mechanism by which VCGs boost anti-chlamydial immune responses through DC activation, we evaluated the ability of BMDCs to internalize VCGs and its effect on DC viability, maturation, and presentation of chlamydial antigen to CD4+ T cells. Our results showed that VCGs are efficiently internalized by DCs without affecting their viability. This is consistent with a previous report showing that human conjunctival epithelial cells have a high capacity to functionally internalize bacterial ghosts without any cytotoxic effects [26].

The present study used UV-EBs with or without VCGs to stimulate DC maturation. The interaction of VCGs with BMDC stimulated the expression of co-stimulatory molecules associated with DC activation and maturation. In addition, there is enhanced expression of major histocompatibility complex molecules (MHC) and these surface molecules mediate adhesion with T cells during antigen presentation [27]. Consistent with previous findings [28], stimulation of BMDCs with UV-EBs for 24 h resulted in the significant up-regulation of costimulatory molecules (CD40, CD80, CD83 and CD86) and class II major histocompatibility complex molecules (MHC II) that was enhanced by co-culture with VCGs. These molecules are well-recognized markers for mature dendritic cells and may be involved in regulation of antigen presentation. We found no change in the expression of the transferrin receptor, CD71 and the monocyte lineage marker CD14 consistent with previous findings indicating stimulation of BMDCs with a VCG-based vaccine resulted in decrease in expression of CD14 and CD71 [29]. CD71 is essential for cellular growth and is usually expressed by immature proliferating cells and at low levels on resting mature lymphocytes. CD14 is expressed by monocytes, macrophages and immature DCs but not by mature DCs [30,31]. The low expression of CD14 and CD71 by VCG-pulsed DCs confirms that these DCs are in a fully activated and mature state. Also, the inability of VCG to induce DCs to express CD14 confirms previous findings by Haselberger et al. [32] indicating that LPS in the intact Gram-negative cellular envelop does not possess the same stimulatory and pyrogenic potential as purified LPS. This will explain the observation that repeated systemic administration of VCG even in very high doses fail to induce pyrogenic effects in experimental animals [29,33].

VCG activation of BMDC enhanced the secretion of proinflammatory cytokines, particularly IL-12 and TNF-α and chlamydial antigen presentation to infection-sensitized CD4+ T cells leading to enhanced proliferation and secretion of Th1-type cytokines. These findings are consistent with the report of a recent study showing that a VCG-based chlamydial vaccine induced enhanced proinflammatory cytokine production by BMDCs [29]. Inflammation is a fundamental defense response, and is associated with the secretion of proinflammatory and anti-inflammatory cytokines. IL-12 is naturally produced by DCs in response to antigenic stimulation, and is critical for directing the differentiation of naïve T cells into Th1 cells [34] and effective protection against genital chlamydial infection [35] due to its role in inducing T cells to produce IFN-γ. The secretion of IL-12 by activated BMDCs suggests a possible mechanism by which VCG-based vaccines enhance CD4+ Th1 immune responses in vivo. VCG also significantly enhanced the proliferation of *Chlamydia*-specific CD4+ T cells and IFN-γ secretion in response to in vitro restimulation with chlamydial antigen as reported previously [20]. Studies of protective immunity in the murine model of *C. trachomatis* genital infection demonstrated that an MHC class II restricted CD4+ Th1 response is critical for resolving primary chlamydial infection [36,37].

Another significant finding of this study is the capacity of VCG to enhance the immunogenicity and protective ability of adoptively administered DC-based cellular vaccine. The results showed that co-delivery of antigen-pulsed DCs with VCG enhanced the chlamydial-specific antibody and T cell responses that conferred protective immunity against challenge. It has been suggested that adjuvants act on DCs possibly by providing danger signals [38] that stimulate cellular activation for an enhanced antigen handling and presentation. Thus, previous studies showed that VCG-based vaccines elicited enhanced cellular and humoral immunity in the absence of external adjuvants [13,20]. The observed adjuvanticity of VCG may be due to its ability to provide danger signals related to the surface characteristics of the *Vibrio* envelope complex. Since the morphology of VCGs is not denatured during ghost production, all major immune stimulating constituents of the envelope [10,39], comprising LPS, Monophosphoryl lipid A (MPL), outer membrane proteins, toxin co-regulated pili, peptidoglycan or flagellin are preserved. Retention of these constituent immunomodulatory pathogen-associated molecular patterns (PAMPs) that are recognized by toll-like receptors (TLRs) contributes to the immunoenhancing potential of VCGs. The *V. cholerae* cell envelope carrier system (VCG) is thus a self-adjuvanting delivery platform naturally endowed with potent adjuvant properties. Besides, VCGs have limited or no toxicity that is commonly associated with the purified LPS of Gram-negative bacteria. This obviates a potential problem in using VCGs as vaccine carriers or adjuvants. In fact, specific studies have shown that VCGs from *V. cholerae* serotype Inaba, exhibited 1000-fold less endotoxin units (EU) per ml than the purified LPS as analyzed by the Limulus Amoebocyte Lysate assay [32]. Also, previous immunologic studies showed that *E. coli*, *Salmonella typhimurium* and *V. cholerae*-derived bacterial ghosts are comparatively less pyrogenic than LPS in rabbits [40]. Therefore, endotoxicity does not limit the use of the *V. cholerae* bacterial ghost system as a vaccine carrier or adjuvant. Moreover, existing and acceptable licensed human cholera vaccines are either attenuated or inactivated whole *V. cholerae* cells with intact living or nonliving gram-negative bacterial cell envelopes, respectively and are nontoxic [41,42].

## Conclusion

In the present work we demonstrated that VCGs are efficiently internalized by DCs without affecting their viability and this interaction stimulated the expression by DCs of co-stimulatory molecules associated with DC maturation and secretion of proinflammatory cytokines. This interaction also led to enhancement of chlamydial antigen presentation to immune CD4+ T cells leading to enhanced T cell proliferation and secretion of Th1-type cytokines. Taken together, these results indicate that the immunomodulatory function of VCG for increased T-cell activation is mediated, at least in part, through DC triggering suggesting that VCGs would be a promising tool as an immunomodulator to enhance type-1 immunity.

## Abbreviations

VCG: *Vibrio cholerae* ghost
DC: Dendritic cells
BMDC: Bone marrow-derived dendritic cells
FACS: Fluorescence-activated cell sorting
PE: Phycoerythrin
FITC: Fluorescein isothiocyanate
UV-EBs: Ultraviolet radiation-treated chlamydial elementary bodies
APC: Antigen presenting cell
IL: Interleukin
IFN: Interferon
TNF: Tumor necrosis factor
CD: Cluster of differentiation
Th: Helper T cells
IMDM: Iscove’s Modified Dulbecco’s Medium
FCS: Fetal calf serum
ME: Mercaptoethanol
GM-CSF: Granulocyte macrophage-colony stimulating factor
PBS: Phosphate buffered saline
BSA: Bovine serum albumin
BrdU: 5-Bromo-2′-deoxy-uridine
Flt3L: Fms-like tyrosine kinase 3 ligand
LPS: Lipopolysaccharide
MHC II: Major histocompatibility complex type II
TLR: Toll-like receptor
MPL: Monophosphoryl lipid A
PAMPs: Pathogen-associated molecular patterns
EU: Endotoxin units
